# Exogenous biological renal support ameliorates renal pathology after ischemia reperfusion injury in elderly mice

**DOI:** 10.18632/aging.101899

**Published:** 2019-04-12

**Authors:** Dong Liu, Zhiwei Yin, Qi Huang, Yi Ren, Diangeng Li, Linna Wang, Shaoyuan Cui, Ying Zhang, Yichun Ning, Lide Lun, Guangyan Cai, Xueyuan Bai, Xuefeng Sun, Xiangmei Chen

**Affiliations:** 1 Department of Nephrology, Chinese PLA General Hospital, Chinese PLA Institute of Nephrology, State Key Laboratory of Kidney Diseases, National Clinical Research Center for Kidney Diseases, Beijing 100853, China; 2 Department of Nephrology, Air Force Medical Center, PLA, the Fourth Military Medical University, Beijing 100142, China; 3 Department of Nephrology, Beijing Tiantan Hospital, Capital Medical University, Beijing 100050, China; 4 Department of Nephrology, Zhongshan Hospital, Fudan University, Shanghai 200032, China

**Keywords:** aged, renal ischemia reperfusion injury, parabiosis model, exogenous biological renal support, cytokines

## Abstract

We established an exogenous biological renal support model through the generation of parabiotic mice. At 72 hours after ischemia reperfusion injury (IRI), the aged mice that received exogenous biological renal support showed significantly higher levels of renal cell proliferation and dedifferentiation, lower levels of renal tubular injury, improved renal function, and a lower mortality than those that did not receive exogenous biological renal support. Using the Quantibody Mouse Cytokine Antibody Array, we found that aged IRI mice that received exogenous biological renal support had an up-regulation of multiple inflammatory related cytokines compared to the group that did not receive exogenous biological renal support. We suggest that the exogenous biological renal support might promote renal tubular epithelial cell proliferation and dedifferentiation and improve the prognosis of aged IRI mice. Exogenous biological renal support may play an important role in the amelioration of renal IRI by regulating the expression of multiple cytokines.

## Introduction

Patients of advanced age are at a high risk of developing acute kidney injury (AKI). Compared to younger patients with AKI, older ones have more severe renal pathology, develop more complications, and have a significantly high mortality [1–5]. Although blood purification techniques, such as continuous renal replacement therapy (CRRT), are available, studies have not demonstrated their beneficial effects for overall or renal survival among AKI patients [5–9]. Conversely, the use of an artificial biological kidney has been shown to reduce mortality among AKI [10,11]. We propose that, compared to artificial biologicalrenal system, blood purification techniques, including CRRT, neither effectively remove circulating renal injury-associated adverse factors, nor provide beneficial effects for facilitating recovery from AKI. Furthermore, which cytokines effectively removed by artificial biological kidney are still unknown, the mechanisms through which the technique decrease mortality among AKI are unclear.

Parabiotic mice are used as an experimental model, which involves the surgical joining of the muscle and hypoderm of two mice, in order to create a shared circulatory system. Blood cells and soluble factors can freely circulate within this shared system continuously at physiological levels. Recently, the parabiotic model has been used to study the effect of a shared circulatory system on organ function and the extent of recovery after injury, based on mice with primary biliary cirrhosis (PBC) gene modifier and wild type mice, this approach successfully ameliorated the murine autoimmune cholangitis [12]. Another parabiosis study connected mice with surgery-induced myocardial infarction and healthy littermates. This study found that blood cells from the healthy animals facilitated the regeneration of injured cardiomyocytes in the diseased mice [13]. Parabiosis has also been shown to alleviate brain and peripheral amyloid-beta potentiation in mice with Alzheimer's disease [14], and to participate in early meniscal healing after injury [15]. The mechanisms through which these effects are exerted involve the achievement of protective immunity between two organs, mediated by soluble factors in the shared circulation [16]. Moreover, the non-injured individual can provide continuous biological renal support to the mice sustaining AKI through parabiosis. On the other hand, the circulating cytokines involved in influencing the prognosis of parabiotic IRI mice are still unclear.

Aging kidneys exhibit increased oxidative stress, inflammation, dysregulated autophagy and mitochondrial homeostasis, and a delayed renal recovery after injury [17]. Elderly with AKI are less likely to recover than the young [18]. Our previous experiments involving parabiosis showed that youthful milieu might attenuate inflammation, apoptosis, and increase autophagy in older mice through the amelioration of renal senescence [19]. The aged mice IRI model that was established based on parabiosis found that youthful systemic milieu might decrease the level of oxidative stress, inﬂammation, and apoptosis, while increasing autophagy and improving the renal pathology after IRI in aged animals [20]. On the other hand, it was found that older mice sustaining renal IRI had a poor renal recovery compared to younger ones with IRI, while cytokines, such as GDF11, improved renal tubular proliferation, dedifferentiation, and the prognosis of renal IRI in elderly mice [21].

In this study, we administered IRI to aged mice, and provided them with exogenous biological renal support through parabiosis, followed by monitoring the mortality, renal function and pathologic changes, including tubular proliferation and dedifferentiation at 72 hours after IRI. Our aim was to identify whether exogenous biological renal support promoted renal recovery from IRI and improved prognosis, and to explore the preliminary mechanisms involved.

## Results

### Exogenous biological renal support ameliorates the acute tubular injury score, lowers serum Cr, BUN, and decreases mortality in the old IRI mice

**Figure 1:**
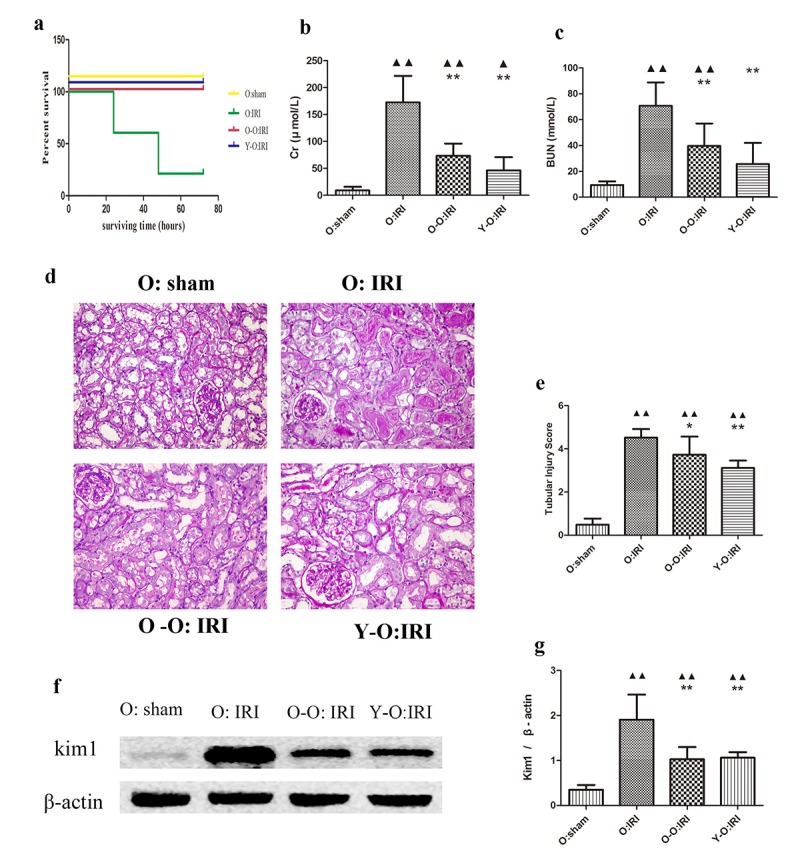
**Exogenous biological renal support improved renal IRI and decreased mortality and serum Cr, BUN levels in old IRI mice.** (**A**) Survival curves for the old IRI mice at 72 hours. (**B**) Cr levels in the old mice. (**C**) BUN levels in the old mice. (**D**) Representative photographs of kidney sections from the old mice stained with periodic acid–Schiff (400× magnification). (**E**) Renal tubular injury score. (**F**) The levels of Kim1 in kidney extracts from the old mice, as measured by western blotting. Gels were performed under the same experimental conditions. (**G**) Quantitative analyses of the band densities of Kim1 expression. Values are presented as means ± SDs. ▲*P* < 0.05, ▲▲*P* < 0.01 vs. O: sham; *P < 0.05, **P < 0.01 vs. O: IRI. BUN, blood urea nitrogen Cr, serum creatinine; SD, standard deviation.

At 72 hours following the procedure, 21.4% of the old IRI mice survived. Conversely, 100% of the old-old parabiotic or the young-old parabiotic IRI mice survived for this time period. At 72 hours following IRI, the old IRI mice showed extensive loss of tubular brush borders, epithelial necrosis and degeneration of the basement membrane, cast formation, and tubular dilatation, accompanied by an increase in the acute tubular injury score. The expression of Kim1, a marker of renal tubular injury, was also elevated, as was serum Cr and BUN. Compared to the old: IRI group, the old parabiotic IRI mice of the O-O: IRI group and the Y-O: IRI group had significantly lower acute tubular injury scores, significantly lower Kim1 expression, and a significantly smaller increase in serum Cr and BUN. There was no significant difference between the aged IRI mice of the old-old parabiotic group and the aged IRI mice of the young-old parabiotic group, with regard to acute tubular injury scores, serum Cr, BUN, and mortality. These findings suggest that, at 72 hours following IRI, the exogenous biological renal support introduced by parabiosis from either an old-old or a young-old pairing could significantly alleviate renal histological injury and improve serum Cr, BUN, and mortality in old IRI mice (Figure 1).

### Exogenous biological renal support promotes tubular cell proliferation in the old IRI mice kidney

**Figure 2:**
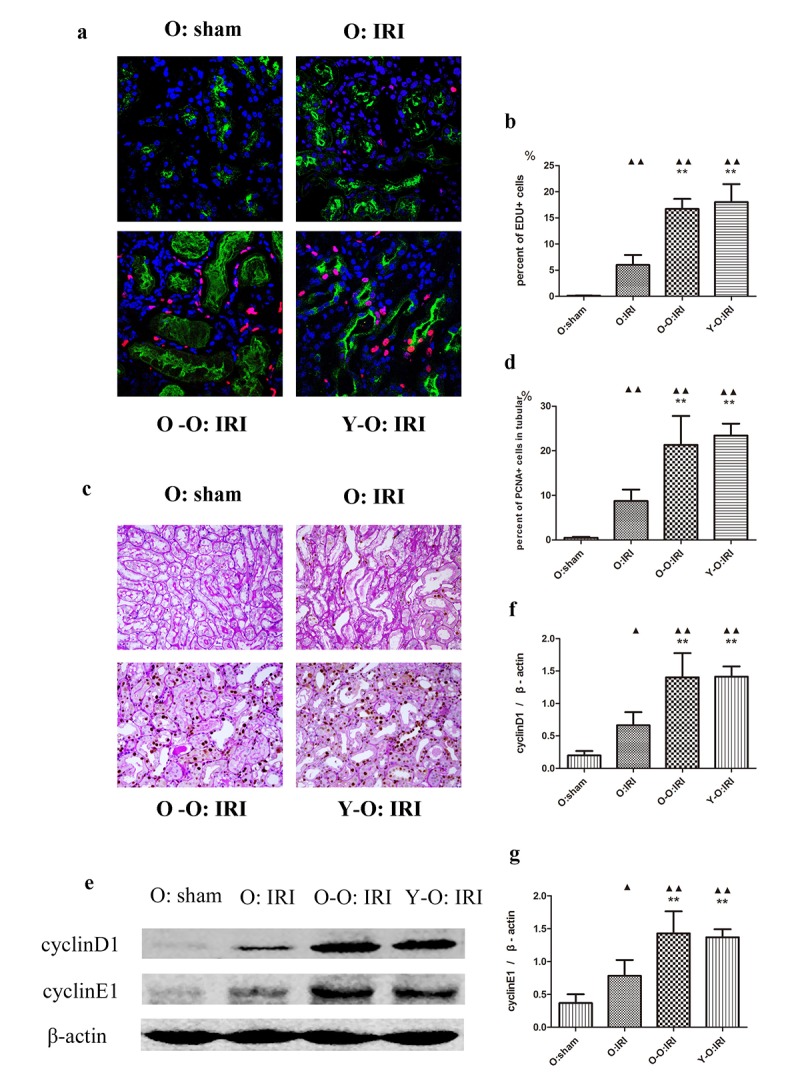
**Exogenous biological renal support increased renal cell proliferation in old IRI mice.** (**A**) Representative images of renal EdU-positive cells in independent groups (600× magnification; red, EDU; green, LTL; blue, DAPI). (**B**) The percentages of EdU-positive cells in the kidneys of the old mice at 72 hours after IRI. The mice in the O: IRI group displayed more EdU-positive cells than in O: sham group. The percentage of EdU-positive cells was higher in the O-O: IRI group and the Y-O: IRI group than in the O: IRI group. No significant difference was found between the O-O: IRI group and the Y-O: IRI group. (**C**) Representative images of renal PCNA-positive tubular cells in independent groups (400× magnification). (**D**) The percentages of PCNA-positive tubular cells in the kidneys of the old mice at 72 hours after IRI. The mice in the O: IRI group had more PCNA-positive tubular cells than the O: sham group. The percentages of PCNA-positive tubular cells were higher in the O-O: IRI group and the Y-O: IRI group than in the O: IRI group. No significant difference was found between the O-O: IRI group and the Y-O: IRI group. (**E**) The levels of cyclin D1 and cyclin E1 in kidney extracts of the old IRI mice as measured by western blotting. Gels were performed under the same experimental conditions. (**F, G**) Quantitative analyses of the band densities of cyclin D1 and cyclin E1 protein expression. Data are presented as means ± SDs. ▲P < 0.05, ▲▲P < 0.01 vs. O: sham; *P < 0.05, **P < 0.01 vs. O: IRI. SD, standard deviation.

At 72 hours following IRI, the percentage of 5-Ethynyl-2-deoxyuridine (EDU)-positive and proliferating cell nuclear antigen (PCNA)-positive cells was higher in the old: IRI group than in the old: sham group. EDU-positive cells were mostly located outside the tubular brush borders, as marked by lotus tetragonolobus lectin (LTL), while PCNA-positive cells were mostly located within the tubular base membranes, as marked by periodic acid–Schiff (PAS) staining. Furthermore, the expression of cyclin D1 and cyclin E1 was also significantly higher in the old: IRI mice than in the old: sham group. In addition, the percentage of EdU-positive cells and PCNA-positive renal tubular cells in the O-O: IRI and the Y-O: IRI group were significantly higher than in the old: IRI group, accompanied by higher cyclin D1 and cyclin E1 expressions. Conversely, there was no significant difference regarding aged IRI mice between the old-old parabiotic group and the young-old parabiotic group. These findings indicate that the exogenous biological renal support provided by parabiosis can significantly improve tubular cell proliferation in old IRI mice kidney (Figure 2).

### Exogenous biological renal support promotes dedifferentiation in the old IRI mice kidney

**Figure 3:**
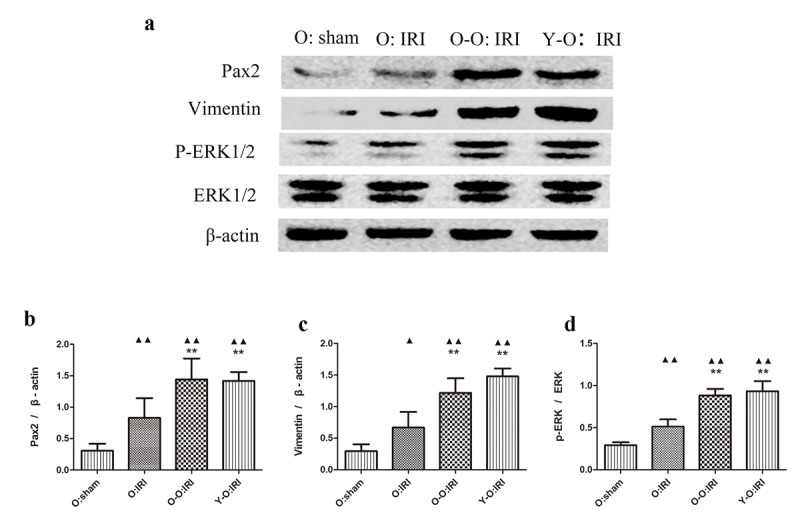
**Exogenous biological renal support increased dedifferentiation in old IRI mice kidney.** (**A**) The levels of Pax2, vimentin, and ERK1/2 in kidney extracts of the old IRI mice as measured by western blotting. Gels were performed under the same experimental conditions. (**B**–**D**) Quantitative analyses of the band densities of Pax2, vimentin, and ERK1/2 expressions. Data are presented as means ± SDs. ▲P < 0.05, ▲▲P < 0.01 vs. O: sham; *P < 0.05, **P < 0.01 vs. O: IRI. SD, standard deviation.

Dedifferentiated tubular cells are primarily responsible for repair after renal IRI, and ERK1/2 may promote tubular dedifferentiation and proliferation, which we examined using vimentin, Pax2, and ERK1/2 [18]. We found that the expressions of vimentin, Pax2 and ERK1/2 was significantly higher in the old: IRI groups 72 hours after IRI, than in the old: sham group. The expression of vimentin, Pax2, and ERK1/2 was significantly higher in the O-O: IRI group and the Y-O: IRI group than the old: IRI group. There was no significant difference regarding these proteins between the O-O: IRI group and the Y-O: IRI group. These findings indicate that the exogenous biological renal support provided by parabiosis may promote renal dedifferentiation in old IRI mice (Figure 3).

### The impact of serum cytokines on the prognosis of the old IRI mice

**Figure 4:**
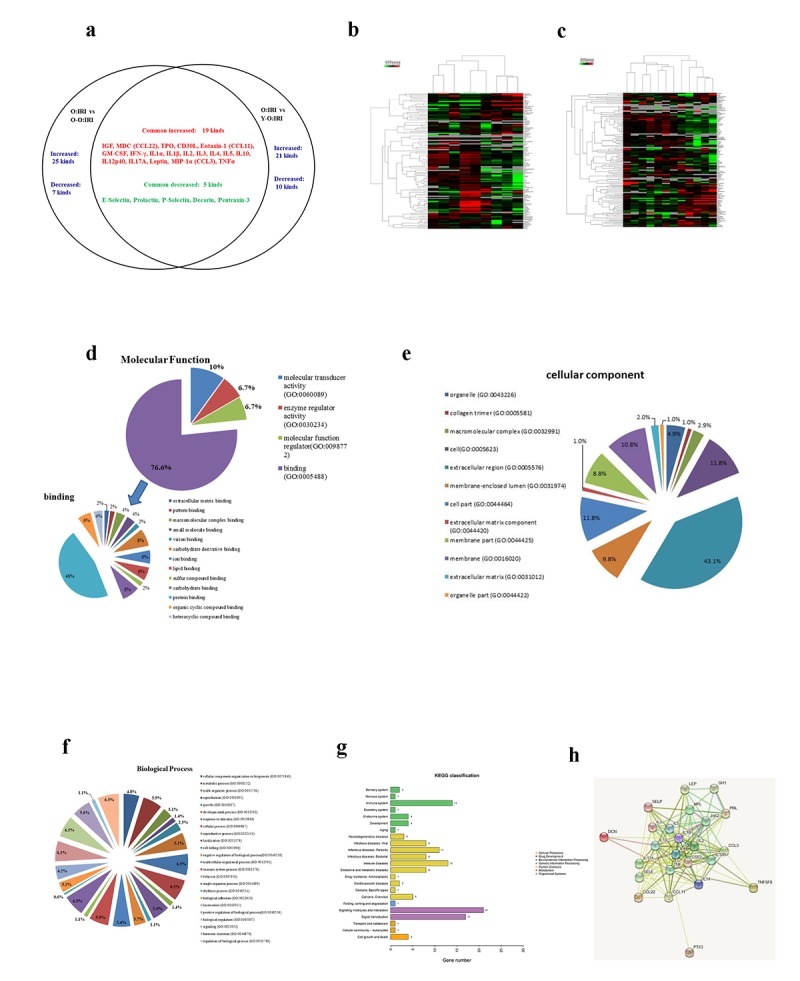
**The impact of serum cytokines on the prognosis of the old IRI mice.** (**A**) The canonical serum cytokines between the O-O: IRI to the old: IRI group and the Y-O: IRI to the old: IRI group. (**B**) Heat map of the O-O: IRI to the old: IRI group. (**C**) Heat map of the Y-O: IRI to the old: IRI group. (**D**) Gene Ontology Annotation was used to identify the molecular function of the differentially expressed cytokines (**E**) Gene Ontology Annotation was used to identify the cellular components of the differentially expressed cytokines (**F**) Gene Ontology Annotation was used to identify the biological process of differentially expressed cytokines (**G**) The KEGG pathways that changed significantly were those related to differentially expressed cytokines. (**H**) The protein-protein interaction network among the differentially expressed cytokines.

The Quantibody Mouse Cytokine Antibody Array 2000 results demonstrate that serum cytokines were differentially expressed at 72 hours after IRI. Compared to the old: IRI group, in the O-O: IRI group the level of 25 cytokines were significantly higher while 7 were significantly lower. Compared to the old: IRI group, in the Y-O: IRI group the level of 21 cytokines was significantly higher while 10 were significantly lower. We observed 19 commonly up-regulated cytokines, including IGF-1, MDC (CCL22), thrombopoietin (TPO), CD30 ligand, eotaxin-1 (CCL11), GM-CSF, IFN-gamma, IL-1alpha, IL-1beta, IL-2, IL-3, IL-4, IL-5, IL-10, IL-12p40, IL-17A, leptin, MIP-1alpha (CCL3), and TNF-alpha, while there were 5 commonly down-regulated cytokines, including E-selectin, prolactin, P-selectin, decorin, and pentraxin-3 (Figure 4A–4C, Table 1).

**Table 1:** Significant different protein identified by cluster analysis.

**Gene name**	**Query**	**Protein**	**Homo sapiens name**	**O-O:IRI/O:IRI ratio**	**Y-O:IRI/O:IRI ratio**
IGF-1	mmu:16000	Insulin-like growth factor 1	GH1 - growth hormone 1	3.081	3.395
MDC (CCL22)	mmu:20299	Macrophage-Derived Chemokine	CCL22 – Chemokine (C-C motif) ligand 22	2.3	2.481
TPO	mmu:21832	Thrombopoietin	MPL - Myeloproliferative leukemia virus oncogene	8.293	18.206
CD30L	mmu:21949	Tumor Necrosis Factor (Ligand) Superfamily, Member 8	TNFSF8 - Tumor necrosis factor (ligand) superfamily, member 8	10.864	8.485
Eotaxin-1 (CCL11	mmu:20292	Eotaxin-1	CCL11 - Chemokine (C-C motif) ligand 11	2.169	2.14
GM-CSF	mmu:12983	Colony Stimulating Factor 2 (Granulocyte-Macrophage)	CSF2 - Colony stimulating factor 2	2.253	2.176
IFN-gamma	mmu:15978	Interferon Gamma	JAK2 - Janus kinase 2	2.984	2.454
IL-1alpha	mmu:16175	Interleukin 1 Alpha	Interleukin 1 A	2.751	2.739
IL-1beta	mmu:16176	Interleukin 1 Beta	Interleukin 1 B	3.243	2.616
IL-2	mmu:16183	Interleukin 2	IL2 - Interleukin 2	2.079	1.633
IL-3	mmu:16187	Interleukin 3	IL3 - Interleukin 3	2.643	2.312
IL-4	mmu:16189	Interleukin 4	IL4 - Interleukin 4	2.544	2.226
IL-5	mmu:16191	Interleukin 5	IL5 - Interleukin 5	3.253	2.393
IL-10	mmu:16153	Interleukin 10	IL10 - Interleukin 10	2.335	1.871
IL-12p40	mmu:16160	Interleukin 12B	IL12B	3.691	3.335
IL-17A	mmu:16171	Interleukin 17A	Interleukin 17A	3.605	2.86
Leptin	mmu:16846	Leptin	LEP – Leptin	4.439	3.575
MIP-1alpha (CCL3)	mmu:20302	Macrophage Inflammatory Protein 1-Alpha	CCL3 - Chemokine (C-C motif) ligand 3	4.173	2.95
TNF-alpha	mmu:21926	TNF-Alpha	TNF - Tumor necrosis factor	2.938	2.59
E-Selectin	mmu:20339	Selectin E	SELE - Selectin E	0.636	0.661
P-Selectin	mmu:20344	Selectin P	SELP - Selectin P	0.597	0.601
Decorin	mmu:13179	Decorin	DCN - Decorin	0.604	0.628
Pentraxin-3(TSG-14)	mmu:19288	Pentraxin 3	PTX3 - Pentraxin 3	0.522	0.248
Prolactin	mmu:19109	Prolactin	PRL - Prolactin	0.015	0.117

Gene ontology (GO) annotation was used to identify the molecular function, cellular component, and biological processes of the involved common differentially expressed cytokines. The molecular function of the proteins involved in binding, molecular transducer activity, molecular function regulator, and the enzyme regulator activity. Moreover, the proteins involved in binding accounted for 76.6% of all proteins. Further analysis revealed protein binding occupying 46% of the binding molecular function (Figure 4D). Through cellular component analysis, we found that the largest proportion of differentially expressed proteins belonged to the extracellular region (43.1%; Figure 4E). Through biological process analysis, we identified the response to stimulus, cellular process, single-organism process, biological regulation and regulation of biological process as the top five categories (Figure 4F). The 24 differentially expressed proteins were categorized into various pathways, including inflammation mediated by chemokine and cytokine signaling pathway, and the interleukin signaling pathway based on the PANTHER classification system (Table 2). To better understand the characteristics of the differentially expressed proteins, the KEGG database was used for pathway analysis, with 23 pathways listed. The pathways with the most significant changes were those related to signaling molecules and interactions, signal transduction (Figure 4G). In these differentially expressed proteins, protein-protein interaction networks were identified using the STRING analysis (Figure 4H).

**Table 2:** Differentially expressed cytokines related pathway by PANTHER analysis.

**Gene name**	**Query**	**Pathway - PANTHER**
IGF-1	mmu:16000	P00033:Insulin/IGF pathway-protein kinase B signaling cascade;
	Igf1	P00032:Insulin/IGF pathway-mitogen activated protein kinase kinase/MAP
MDC	mmu:20299	P00031:Inflammation mediated by chemokine and cytokine signaling pathway
(CCL22)	Ccl22	
TPO	mmu:21832	
	Thpo	
CD30L	mmu:21949	
	Tnfsf8	
Eotaxin-1	mmu:20292	P00031:Inflammation mediated by chemokine and cytokine signaling pathway
(CCL11)	Ccl11	
GM-CSF	mmu:12983	P00036:Interleukin signaling pathway
	Csf2rb	
IFN-gamma	mmu:15978	P00035:Interferon-gamma signaling pathway;
	Ifng	P00031:Inflammation mediated by chemokine and cytokine signaling pathway
IL-1alpha	mmu:16175	P00036:Interleukin signaling pathway
	Il1a	
IL-1beta	mmu:16176	P00031:Inflammation mediated by chemokine and cytokine signaling pathway
	Il1b	
IL-2	mmu:16183	P00031:Inflammation mediated by chemokine and cytokine signaling pathway;
	Il2	P00036:Interleukin signaling pathway
IL-3	mmu:16187	P00036:Interleukin signaling pathway
	Il3	
IL-4	mmu:16189	P00036:Interleukin signaling pathway
	Il4	
IL-5	mmu:16191	P00031:Inflammation mediated by chemokine and cytokine signaling pathway;
	Il5	P00036:Interleukin signaling pathway
IL-10	mmu:16153	P00036:Interleukin signaling pathway
	Il10	
IL-12p40	mmu:16160	
	Il12b	
IL-17A	mmu:16171	P00031:Inflammation mediated by chemokine and cytokine signaling pathway;
	Il17a	P00036:Interleukin signaling pathway
Leptin	mmu:16846	
	Lep	
MIP-1alpha(CCL3)	mmu:20302	P00031:Inflammation mediated by chemokine and cytokine signaling pathway
	Ccl3	
TNF-alpha	mmu:21926	P00057:Wnt signaling pathway;
	Tnf	P00006:Apoptosis signaling pathway
E-Selectin	mmu:20339	
	Sele	
P-Selectin	mmu:20344	
	Selp	
Decorin	mmu:13179	
	Dcn	
Pentraxin-3(TSG-14)	mmu:19288	
	Ptx3	
Prolactin	mmu:19109	
	Prl	

## Discussion

The parabiosis model creates a shared circulation between two mice, allowing the two animals to exchange blood cells and soluble factors. A previous study has demonstrated that there is a mean exchange flow of 0.66% of the circulating blood volume among the parabiotic mice per hour [22]. This exchange flow is equivalent to a mean daily exchange of 8% of the circulating volume. This exchange results in an equal distribution of blood cells in each parabiont [22]. In this study, after the parabiosis was established between EGFP transgenic mice and wild-type mice, rare GFP-positive cells were detected in the kidneys of the wild-type mice. This indicated that the beneficial effect on aged IRI mice in the parabiosis model were unlikely to result from exogenously transferred blood cells. The non-injured mice might provide exogenous biological renal support to the IRI mice in the parabiotic model. Through the shared circulation, the non-injured mice partially assisted in water excretion, metabolism, and the endocrine function for the injured mice. The effects of cytokine regulation by the exogenous biological renal support played an important role.

Renal IRI is one of the most common causes of AKI. Triggered by oxidative stress and inflammation, renal tubular epithelia undergo apoptosis, necrosis, and proliferation repair [23,24]. Researchers have found that oxidative stress, inflammation, and apoptosis in multiple organs increase significantly in the elderly [2], and aged patients with AKI sustain more severe injury and poor renal recovery [25,26]. In our previous study, using the same parabiosis mice model, we observed a lower level of oxidative stress, inflammation, and apoptosis and increased autophagy with a decreased tubular injury score in the Y-O parabiosis IRI group compared with the O-O parabiosis IRI group at 24 hours after IRI [20]. In the current study, at 72 hours after IRI, the renal tubular injury score did not differ significantly between the Y-O parabiosis IRI group and the O-O parabiosis IRI group. However, both parabiosis groups had injury scores lower than those of the old IRI mice. We propose that the youthful systemic milieu ameliorated renal injury at 24 hours after IRI in the aged mice, but not at 72 hours. Furthermore, the exogenous biological renal support from either the younger kidney or the older kidney improved renal injury in the aged mice at 72 hours after IRI. The injured kidneys were considered to enter into the recovery stage at 72 hours after IRI [21]. These results indicated that exogenous biological renal support promoted renal recovery after injury; consequently, the beneficial influence from the youthful systemic milieu was less prominent at this time.

Renal function recovery is histologically characterized by newer renal tubular cells replacing those sustained injuries [27]. Many signaling pathways related to systemic milieus take part in this recovery process. The pathways that have been shown to affect AKI repair response include inflammatory [28], autophagy [29], soluble cytokines and growth factors [30], mesenchymal stem cells [31], immune cells [32], extracellular vesicles (EVs) [33]. It has recently been shown that mature renal tubular epithelial cells could migrate and dedifferentiate, followed by proliferation to restore renal tubular structures [34,35]. Differentiation plays an important role in the process of proliferation [36]. Our previous study showed that aged IRI mice had a decreased level of renal recovery than younger mice, and that GDF11 promoted renal tubular proliferation, differentiation, and improved prognosis [21]. In this study, the parabiotic old IRI mice had a significantly higher number of EDU positive cells and PCNA positive tubular cells, and had an increased expression of cyclin D1, cyclin E1, Pax2, vimentin, and ERK1/2 but a decreased acute tubular injury score and a lower expression of Kim1, serum BUN, and Cr level, while their survival improved significantly compared to the results observed in the old IRI mice. These findings suggest that the exogenous biological renal support provided by the shared circulation likely removes harmful cytokines from the old IRI mice, and/or supplies beneficial cytokines to the host, leading to less severe renal injury, more tubular proliferation and dedifferentiation, and a better prognosis. Furthermore, the effects posed by the systemic internal environment of the young or the old mice might be poorer than those posed by the exogenous biological renal support.

It is still unclear which harmful cytokines were removed by the exogenous biological renal support and/or which protective cytokines were supplied. However, the differences in the levels of cytokines did improve the survival of the old IRI mice. To address this issue, we used a cytokine array to screen for differentially expressed cytokines in the mice serum. The results were as follow: (1) the cytokines that were differentially expressed cytokines between the Y-O:IRI and O:IRI groups were similar to those that differed between the O-O:IRI and O:IRI groups. This suggests that there is no significant difference in the effect posed by the circulating internal environment between the younger mice and the older mice. (2) the common differentially expressed cytokines between the Y-O:IRI and O:IRI groups and between the O-O:IRI and O:IRI groups were identified. Most of these differentially expressed proteins are involved in biological pathways related to inflammation mediated by chemokines and cytokine signaling pathways, and the interleukin signaling pathway using PANTHER analysis. Using KEGG pathway analysis, the proteins related to signaling molecules and interactions, and signal transduction were identified. Furthermore, the majority of these cytokines belonged to the protein-protein interaction network. This indicates that an interaction occurs between these cytokines. These results suggest that exogenous biological renal support may up-regulate inflammatory related factors, which play an important role in the amelioration of renal IRI and the lowering of the mortality.

A previous study reported that blocking the release of IL-22 during the early injury phase protects animals from renal damage, while IL-22 blockade attenuates tubular repair at day 5 after ischemic renal injury. Furthermore, restoration of IL-22 in these animals accelerated tubular regeneration and the recovery after AKI [37]. Our previous study using the parabiotic mice model showed that 24 hours after IRI, compared with the results from the O-O:IRI group, aged mice in the Y-O:IRI group exhibited lower levels of oxidative stress and inflammation, with lower levels of renal injury [20]. In the current study, at 72 hours after IRI, the exogenous biological renal support up-regulated the levels of multiple circulatory inflammatory cytokines, increased renal cell proliferation and dedifferentiation, and improved prognosis compared to the results of animals that did not receive exogenous renal support. Inflammation may exhibit a dual influence on the course of injury and recovery after AKI [38]. Therefore, we suspect that regardless of disease stages, non-selective removal of inflammatory mediators by the blood purification technique might disturb renal recovery after IRI. However, this idea still needs further verification.

In summary, using a parabiotic mice model, we demonstrated that exogenous biological renal support ameliorates renal pathology and decreases the mortality in old IRI mice. We also found that certain serum cytokines influence renal recovery after ischemic reperfusion injury. This study will provide new information for the treatment of AKI in the elderly.

## Materials and Methods

### Experimental animals

The Animal Ethics Committee of the Chinese PLA General Hospital and Military Medical College approved all animal protocols. We purchased 12-week-old male C57BL/6 mice and 24-month-old male C57BL/6 mice from the Si Bei Fu Laboratory Animal Company (Beijing, China). We also bought 12-week-old male C57BL/6-TgN (ACTbEGFP) transgenic mice (GFP+) from the Model Animal Research Center of Chinese Nanjing University (Nanjing, China); mice were kept until 24-month-old in the Si Bei Fu Laboratory Animal Company (Beijing, China).

Parabiosis was performed based on the approach devised by Donskoy and Goldschneider [22], with shared circulation created between two mice verified [20]. After 3 weeks of parabiosis, these mice received bilateral renal IRI as previously described [39]. In brief, we clamped both renal pedicles for 28 minutes with a microvascular clamp (Harvard Apparatus, 728816), followed by clamp removal and visually-confirmed reperfusion. Both healthy and parabiotic mice were given 5 mg/kg 5-Ethynyl-2′-deoxyuridine (EdU) after IRI intraperitoneally of the same dose. Blood and kidneys were harvested from the older mice 72 h after IRI.

Older mice were divided into 4 groups randomly: (1), old sham group (O:sham), 6 pieces 24-month-old mice, who underwent surgery to expose the bilateral renal pedicles without clamping; (2), old IRI group (O:IRI), 6 pieces 24-month-old mice, who received bilateral renal pedicle clamping; (3), old-old parabiotic IRI group (O-O:IRI), 12 pieces 24-month-old mice, who received parabiosis creation initially, followed by bilateral renal pedicles clamping 3 weeks later in an older mice; and (4), young-old parabiotic IRI group (Y-O:IRI), 7 pieces 3-month-old mice and 7 pieces 24-month-old mice, who received parabiosis creation initially, followed by bilateral renal pedicles clamping 3 weeks later in older mice.

### Serum biochemistry analysis

All serum samples were obtained, centrifuged, and stored at -80 °C. We analyzed serum creatinine (Cr) and urea nitrogen (BUN) using an auto-analyzer (Cobas8000, Roche, Germany).

### Histopathological examination

Paraffin-fixed sections were stained with periodic acid–Schiff (PAS), and pathologic examinations were performed by interpreters that were blind to patient identities. We examined pathologic characteristics including cell necrosis, loss of brush borders, cast formation, and tubular dilatation. We scored tubular injury semiquantitatively using a scale of 0–5, where 0, 1, 2, 3, 4, and 5 represents normal findings, ≤ 10%, 11–25%, 25–50%, 50–75%, and > 75%, respectively, based on previous studies [40,41]. We evaluated 10 random non-overlapping fields per animal for scoring [41].

### EdU staining

EdU staining was performed according to the protocol of the Click-iT^®^ EdU Imaging Kit (Life Technologies). We obtained frozen renal sections from old IRI mice; after being fixed with 4% paraformaldehyde and placed with 0.5% Triton X-100 for 5 min, sections were incubated with Click-iT^®^ reaction mixture for 30 minutes. Sections were subsequently immersed with (Fluorescein isothiocyanate; FITC)-conjugated anti-LTL (FL-1321; Vector Labs; 1:1,000) at room temperature for 1 hour, with nuclei counterstained by DAPI. Finally, we used a confocal microscopy (Nikon C1 Eclipse; Nikon) and a standard fluorescence microscopy (Nikon Eclipse 90i; Nikon) to observe the stained sections, and quantified the percentage of EdU-positive cells.

### PCNA immunohistochemistry

We performed antigen retrieval from sections by microwaving them for 10 minutes in 10 mM sodium citrate buffer (pH 6.0), incubated them in 3% hydrogen peroxide for 30 minutes, and placed them in 1.5% normal goat serum for 40 minutes. Sections were subsequently incubated in PCNA (Abcam, 18197, 1:1000) at 4 °C overnight, followed by immersion in biotin-conjugated goat anti-rabbit IgG for 40 minutes, and finally in streptavidin-conjugated peroxidase for another 30 minutes. We also added 3,3′ diaminobenzidine and performed PAS staining. We observed the processed sections under a light microscopy, with percentages of PCNA-positive tubular cells quantified.

### Western blot analysis

Frozen kidney tissues were lysed and centrifuged to obtain the supernatant, followed by proteins separation using sodium dodecyl sulfate-polyacrylamide gel electrophoresis (SDS-PAGE). We transferred proteins to nitrocellulose (NC) membranes and probed the membranes with primary antibodies against the following proteins overnight at 4 °C, including beta-actin (Beijing Biosynthesis Biotechnology Co. 0061R; 1:2000), Pax2 (Proteintech, 21385; 1:800), vimentin (Abcam ab8069; 1:1,000), Kim1(R&D Systems, AF1817; 1:500), ERK1/2 (CST, #9102; 1:800), p-ERK1/2 (CST, #9101; 1:800), cyclin D1 (Proteintech 60186; 1:1,000), and cyclin E1 (Proteintech 11554; 1:1,000). Finally, blots were probed with horseradish peroxidase-conjugated IgG (Santa Cruz Biotechnology, sc-2096; 1:1000), with bands visualized by enhanced chemiluminescence, and quantified densitometrically using the Quantity One software (Bio-Rad Laboratories).

### Quantification of mouse cytokine using antibody array

We collected peripheral blood and centrifuged the specimens, storing serums at -80 °C, followed by protein extraction. We measured 120 cytokines quantified by the Quantibody Mouse Cytokine Antibody Array 2000 (QAM-CAA-2000-4, RayBiotech, Inc., Norcross, GA, U.S.A) according to product instructions. A strict cutoff value is a fold change higher than 1.5 or lower than 0.67, with a P value lower than 0.05. We analyzed proteomic results and visualized them with a heat map using the Cluster and TreeView softwares (http://rana.lbl.gov/EisenSoftware.htm).

### Statistical analyses

All data were analyzed using the SPSS Statistics 13.0 software. Data are expressed as means ± standard deviations (SDs). Statistical analyses were carried out by analysis of variance (ANOVA) followed by appropriate *post hoc* tests, including multiple comparison tests (least significant difference). Cumulative survival were carried out by an analysis of the log rank test (Kaplan–Meier). *P*-values < 0.05 were considered statistically significant.
